# Disrupted Association of Sensory Neurons With Enveloping Satellite Glial Cells in Fragile X Mouse Model

**DOI:** 10.3389/fnmol.2021.796070

**Published:** 2022-01-04

**Authors:** Oshri Avraham, Pan-Yue Deng, Dario Maschi, Vitaly A. Klyachko, Valeria Cavalli

**Affiliations:** ^1^Department of Neuroscience, Washington University School of Medicine, St. Louis, MO, United States; ^2^Department of Cell Biology and Physiology, Washington University School of Medicine, St. Louis, MO, United States; ^3^Hope Center for Neurological Disorders, Washington University School of Medicine, St. Louis, MO, United States; ^4^Center of Regenerative Medicine, Washington University School of Medicine, St. Louis, MO, United States

**Keywords:** satellite glial cells, fragile X syndrome, sensory neuron, neuron-glia communication, hyperexcitability

## Abstract

Among most prevalent deficits in individuals with Fragile X syndrome (FXS) is hypersensitivity to sensory stimuli and somatosensory alterations. Whether dysfunction in peripheral sensory system contributes to these deficits remains poorly understood. Satellite glial cells (SGCs), which envelop sensory neuron soma, play critical roles in regulating neuronal function and excitability. The potential contributions of SGCs to sensory deficits in FXS remain unexplored. Here we found major structural defects in sensory neuron-SGC association in the dorsal root ganglia (DRG), manifested by aberrant covering of the neuron and gaps between SGCs and the neuron along their contact surface. Single-cell RNAseq analyses demonstrated transcriptional changes in both neurons and SGCs, indicative of defects in neuronal maturation and altered SGC vesicular secretion. We validated these changes using fluorescence microscopy, qPCR, and high-resolution transmission electron microscopy (TEM) in combination with computational analyses using deep learning networks. These results revealed a disrupted neuron-glia association at the structural and functional levels. Given the well-established role for SGCs in regulating sensory neuron function, altered neuron-glia association may contribute to sensory deficits in FXS.

## Introduction

Fragile X syndrome (FXS) is the most common heritable cause of intellectual disability and the leading monogenetic cause of autism spectrum disorders (ASD). This condition stems from loss of fragile X mental retardation protein (FMRP), which regulates a wide range of neuronal functions *via* translational control and protein-protein interactions. Some of the most prevalent symptoms of FXS include hypersensitivity to a wide range of sensory stimuli, such as tactile, auditory, and visual stimuli (Cascio, 2010). Sensory hypersensitivity may contribute or even cause behavioral deficits such as anxiety and impaired social interactions (Rais et al., 2018). These sensory deficits have been thus far largely attributed to alterations in sensory processing abnormalities in brain circuits (Contractor et al., 2015). Yet the mechanisms of sensory deficits in FXS and ASD remain elusive. Recent studies suggest that many core cognitive deficits in ASD may arise from earlier deficits in sensory inputs that subsequently drive abnormal development of cortical circuits (Orefice et al., 2016, 2019). Whether and how dysfunction of peripheral sensory system that receive and convey primary sensory information contribute to FXS pathophysiology remains largely unexplored.

In the accompanying manuscript we described a state of profound hyperexcitability of primary sensory neurons in the dorsal root ganglia (DRG) driven, at least in part, by intrinsic neuronal deficits in HCN channels. In addition to neuronal intrinsic mechanisms, glial cells can modulate neuronal structure and function, and are implicated in many neurodevelopmental diseases (Kim et al., 2020). In the brain, astrocytes express FMRP and accumulating evidence suggests widespread alterations in astrocyte-neuronal communication in FXS models (Pacey and Doering, 2007; Jacobs and Doering, 2010; Jacobs et al., 2012, 2016; Yang et al., 2012; Pacey et al., 2015; Cheng et al., 2016; Sourial and Doering, 2016; Wallingford et al., 2017; Krasovska and Doering, 2018). Moreover, astrocyte-selective loss of FMRP contributes to cortical synaptic deficits in FXS through the dysregulated astroglial glutamate transporter GLT1 and impaired glutamate uptake (Higashimori et al., 2016). In the peripheral sensory circuit, satellite glial cells (SGCs) share many functional similarities with astrocytes despite a great diversity in morphology (Avraham et al., 2020, 2021b; Hanani and Spray, 2020; Hanani and Verkhratsky, 2021). As many as 8–10 SGCs completely envelop each DRG neuron (Pannese, 1964). SGCs contribute to abnormal neuronal hyperexcitability in many pain syndromes (Hanani and Spray, 2020) and we recently showed that these cells also contribute to promote peripheral axon regeneration (Avraham et al., 2020, 2021b). While contribution of astrocytes to synaptic and neuronal dysfunction in the brain of FXS models has received a lot of recent attention, the potential contributions of SGCs in FXS remains unexplored.

In this study we combined ultrastructural analyses and single-cell RNAseq to examine changes in sensory neuron association with their enveloping SGCs caused by FMRP loss.

## Results

### Structural Changes in Sensory Neuron-Satellite Glial Cell Association in *Fmr1* KO Mice

**FIGURE 1:**
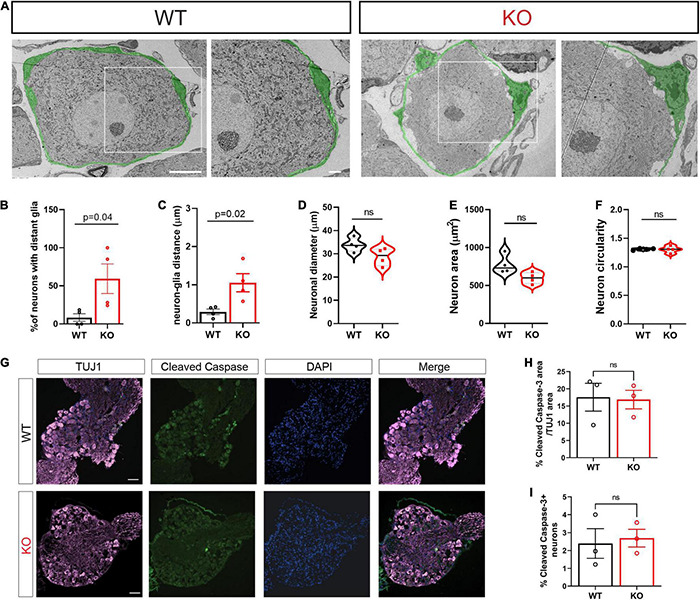
Structural alterations in neuron-SGC association in *Fmr1* KO DRGs with no changes in neuronal morphology. **(A)** Representative TEM images of a neuron-SGC unit in DRG sections from WT and *Fmr1* KO. In total, 4 biologically independent animals. Scale bar: 10 μm (left panel), 2 μm (right panel). The SGCs surrounding sensory neurons are pseudo-colored in green. **(B)** Quantification of percentage of neurons which have gaps between the neuron membrane and the covering SGC, out of the total number of neurons imaged. *n* = 4 biologically independent animals. **(C)** Measurements of the distance between the neuron membrane and the covering SGC membrane (μm). In total, 3 measurements were taken from each neuron. *n* = 4 biologically independent animals. **(D–F)** Measurements of neuronal diameter **(D)**, neuronal area **(E)**, and neuronal circularity **(F)** [ratio between major axis (X) and minor axis (Y). 1 = circular, 1 > elliptic]. *n* = 4 biologically independent animals. **(G)** Representative images of DRG sections co-stained with TUJ1 (magenta) and the apoptotic marker Cleaved Caspase3 (green). *n* = 3 biologically independent animals. Scale bar: 100 μm. **(H)** Quantification of percentage of Cleaved Caspase 3 fluorescence intensity normalized to TUJ1 intensity. *n* = 3 biologically independent animals. **(I)** Quantification of the percentage of cleaved caspase 3 positive neurons out of all neurons per section. *n* = 3 biologically independent animals.

In adult animals, SGCs tightly enwrap the soma of each sensory neuron (Pannese, 1964). The gap between the two cell surfaces can often be as small as ∼20–50 nm. This close association between the two cell types is essential for efficient mutual neuron–SGC interactions (Hanani and Spray, 2020), although the mechanisms regulating and maintaining this close association remain poorly understood. We thus determined the impact of FMRP loss on the structural organization of SGCs surrounding sensory neurons. Transmission Electron Microscopy (TEM) demonstrated the tight contact between SGCs and neurons in WT control mice, whereas the *Fmr1* KOs presented an aberrant covering of the neuron with multiple large gaps between the glia and the neuron along their contact surface (Figure 1A, all values and statistical information is provided in Supplementary Table 1). We found a significant increase in the number of neurons with large gaps along neuron-glia contact surface in the *Fmr1* KO mice compared to WT (Figure 1B). Quantification of the distance between each neuron and the SGC coat further revealed significant defects in the *Fmr1* KO mice with a marked increase in the neuron-glia distances (Figure 1C). However, other aspects of neuronal morphology were similar between *Fmr1* KO and WT mice, with no detectable changes in neuronal diameter, area, and shape (Figures 1D–F). Additionally, immunostaining of sections of DRG from *Fmr1* KO and WT mice for the apoptotic marker cleaved caspase 3 revealed no change in apoptotic cell death in *Fmr1* KO compared to WT mice (Figures 1G–I), indicating that loss of FMRP does not cause excessive neuronal apoptotic cell death. These observations suggest that loss of FMRP results in a failure of SGCs to envelop the neurons tightly. Such a major structural defect might disrupt the neuron-SGC communication. Whether all sensory neurons or only specific subtypes present this aberrant interaction with their surrounding SGC remains to be determined.

### Transcriptional Changes in Dorsal Root Ganglia Cells Caused by Loss of Fragile X Mental Retardation Protein

**FIGURE 2:**
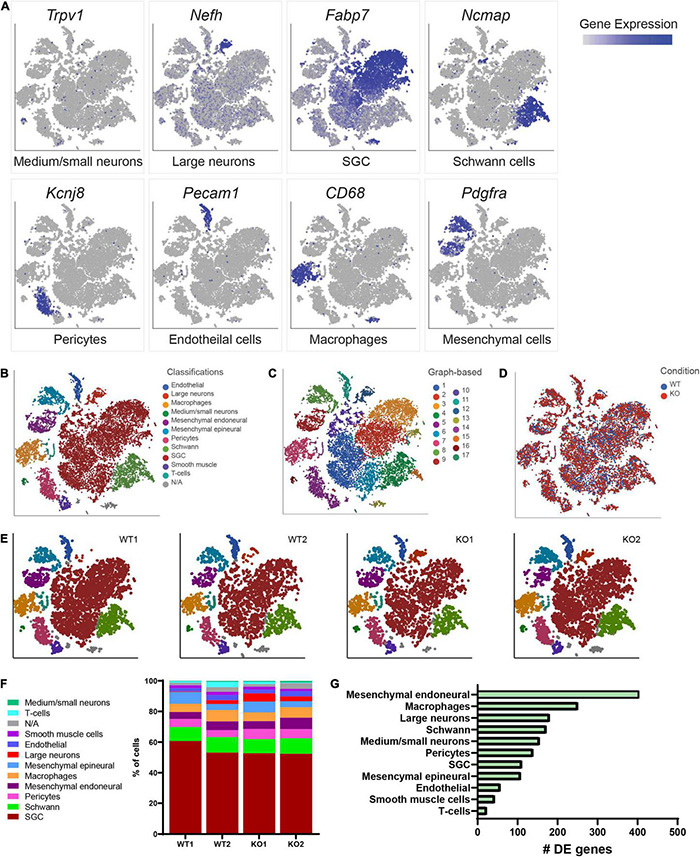
Cluster analysis from scRNAseq of DRGs. **(A)** t-SNE overlay for expression of marker genes for different cell populations in all cells from WT and *Fmr1* KO including Trpv1 for small/medium neurons, Nefh for large neurons, Fabp7 for SGCs, Ncmap for Schwann cells, Kcnj8 for pericytes, Pecam for endothelial cells, CD68 for Macrophages and Pdgfra for mesenchymal cells. The relative levels of expression are presented as a blue color gradient on the left. *n* = 3 biologically independent animals. **(B)** t-SNE plot of 18,000 cells from *Fmr1* KO and WT dissociated DRG. Classifications were assigned based on known marker genes. Low quality cells (3,916 cells from a total of 21,916) with less than 600 genes per cell and doublets were filtered out from analysis (see filtering criteria in the section “Materials and Methods”). In total, 11 distinct cell clusters were assigned based on known marker genes. **(C)** Unbiased, Graph based clustering with 17 distinct cell clusters. **(D)** t-SNE plot colored by genotype. **(E)** t-SNE plots separated by biological sample in WT and KO, colored by cell type. **(F)** Fraction of each cell type within WT (8,970 cells), and *Fmr1* KO (9,038 cells) conditions. **(G)** Plot of the number of differentially regulated genes in each cell type in *Fmr1* KO compared to WT (FDR ≤ 0.05, fold-change ≥ 1.5).

To begin to define the changes in the molecular profile of sensory neurons and SGCs in *Fmr1* KO mice associated with the structural alterations we observed above, we performed single-cell RNA-seq (scRNAseq) from the DRGs of *Fmr1* KO and WT, using the Chromium Single Cell Gene Expression Solution (10x Genomics). This method allows characterization of DRG cells at the molecular level, as described previously (Avraham et al., 2020, 2021b). While scRNAseq captures transcriptional events, changes in RNA stability may also contribute to the different profiles obtained, and the depth of sequencing obtained in scRNAseq analyses might not allow to capture low level transcripts. The number of sequenced cells in *Fmr1* KO mice was 11,060 from 2 biological replicates with an average of 105,076 mean reads per cell, 2,141 mean genes per cell and a total of 20,219 genes detected. The number of sequenced cells from WT mice was 10,856 from 2 biological replicates with an average of 94,139 mean reads per cell, 2,239 mean genes per cell, and a total of 20,066 genes detected. Low quality cells with less than 600 genes per cell and doublets were filtered out from downstream analysis (see filtering criteria in the section “Materials and Methods”). To identify cluster-specific genes, we calculated the expression difference of each gene between that cluster and the average in the rest of the clusters (ANOVA fold change threshold > 1.5). Examination of the cluster-specific marker genes in t-distributed stochastic neighbor embedding (t-SNE) plot, revealed major cellular subtypes including medium/small neurons (*Trpv1*), large neurons (*Nefh/*Nf200), SGCs (*Kcnj10*, *Fabp7*), Schwann cells (*Ncmap*), pericytes (*Kcnj8*), endothelial cells (*Pecam/*Cd31), macrophages (*Cd68*), and mesenchymal cells (*Pdgfra*) (Figures 2A,B and Supplementary Table 2). An unbiased (Graph-based) clustering identified 17 distinct cell clusters (Figure 2C). We then compared the cell clustering between WT and *Fmr1* KO DRGs and found that unique cell clusters were not altered by loss of FMRP (Figure 2D), with similarity in cell cluster distribution between samples and genotypes (Figures 2E,F). To uncover the transcriptional changes that occur within DRG cell clusters in *Fmr1* KO compared to WT control, we calculated the differentially expressed (DE) genes for every cluster (FDR ≤ 0.05, FC ≥ 1.5) and found that loss of FMRP induced substantial gene expression changes in many cell types in the DRG (Figure 2G and Supplementary Tables 3–13).

### Delayed/Aberrant Sensory Neuron Maturation Caused by Loss of Fragile X Mental Retardation Protein

The number of neurons recovered in our scRNAseq analysis was ∼3%, whereas neurons represent ∼12% of cells in the DRG (Avraham et al., 2021b). The relatively low number of neurons recovered might be due to the fact that sensory neurons are relatively large cells, which might be destroyed when passed through microfluidic device and to the potential neuronal damage during tissue dissociation process (Avraham et al., 2020). However, we collected a sufficient number of neurons (248 in WT and 659 in KO) that allowed comparison between genotypes. Since in the accompanying paper we found significant changes in excitability of small/medium *Fmr1* KO neurons, we pooled all cells classified as small/medium neurons (cluster 16; 139 cells in WT and 304 in KO). According to the classification of adult DRG neurons at the single cell level (Sharma et al., 2020), small/medium neurons in our data set included CGRP-α neurons (*Avpr1a, Slc6a7*), CGRP-γ neurons (*Ctcflos, Greb1l*), C-LTMR (*Cacna1i*), Trmp8 neurons (*Trm8*), and excluded proprioceptors (*CGRP*θ, ζ and ε), Aβ LTMR and Aδ LTMR neurons (Supplementary Table 2).

**FIGURE 3:**
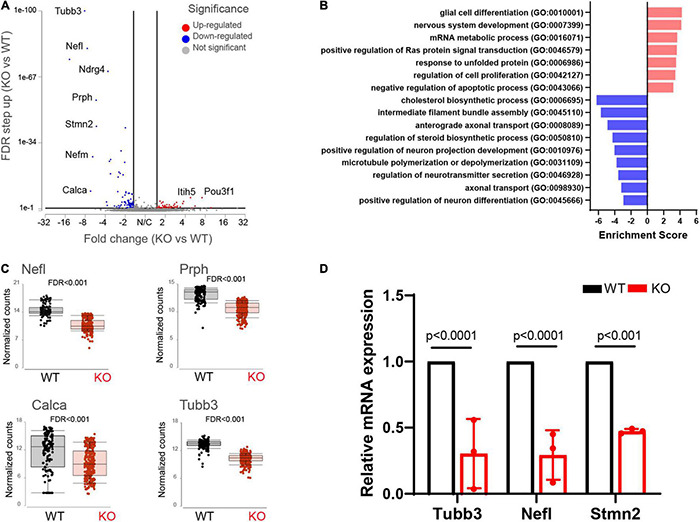
Molecular changes in *Fmr1* KO small/medium sensory neurons indicate delayed differentiation. **(A)** Volcano plot of DE genes in small/medium neurons from *Fmr1* KO compared to WT. **(B)** Enriched pathways in *Fmr1* KO compared to WT (GO Biological Process) for up-regulated (red) and down-regulated (blue) genes. **(C)** Mean expression of the neuronal markers *Nefl*, *Prph*, *Tubb3*, and *Calca* in WT (black) and *Fmr1* KO (red). **(D)** qPCR analysis of the relative mRNA expression of *Tubb3*, *Nefl*, and *Stmn2* in *Fmr1* KO compared to WT DRGs. *n* = 3 biologically independent animals.

We found 155 differentially expressed (DE) genes in the *Fmr1* KO small/medium neurons compared to WT (Figure 2G). In total, 61 genes were upregulated and 94 genes were downregulated (FDR ≤ 0.05 FC ≥ 1.5) (Figure 3A and Supplementary Table 3). We next performed pathway enrichment analysis to reveal the biological function underlying the molecular changes in the *Fmr1* KO neurons (Figure 3B, GO biological process). The upregulated genes were related to nervous system development, cell proliferation, glial cell differentiation and mRNA metabolism (Figure 3B and Table 1). Downregulated genes were related to neuronal differentiation, microtubule and intermediate filament cytoskeleton, axonal transport, neurotransmitter secretion and cholesterol and steroid biosynthesis (Figure 3B and Table 1). Among the transcriptional changes associated with FMRP loss are notable alterations in genes regulating neuronal differentiation and elaboration. Both peripheral neurons and glia arise from neural crest cells. Upon differentiation, sensory neurons acquire neuronal identity by upregulating neuronal markers. Neuronal differentiation markers such as *Ntrk1*; *Ndrg4*; *Avil*; *Stmn2*and markers of axonal/dendritic elaboration such as *Nefl*, *Prph*, *Tubb3*/TUJ1, and *Calca*/CGRP were down regulated in *Fmr1* KO small/medium neurons (Figures 3A,C). We validated these findings by qPCR experiments, which confirmed that the neuronal markers *Tubb3*, *Stmn2*, and *Nefl* were downregulated in *Fmr1* KO DRG compared to WT DRG (Figure 3D).

**TABLE 1:** Enriched biological processes (GO 2018) for differentially up and down regulated genes in *Fmr1* KO small/medium neurons.

GO biological process term	Genes
**Up regulated**	
Glial cell differentiation (GO:0010001)	RELN; ERBB3; SOX10
Nervous system development (GO:0007399)	EDNRB; RELN; ERBB3; B3GNT5; FABP7; NAB2; VLDLR; P OU3F2
mRNA metabolic process (GO:0016071)	SON; ZFP36L2; HSPA1B; HSPA1A
Positive regulation of Ras protein signal transduction (GO:0046579)	COL3A1; NOTCH1; LPAR1
Response to unfolded protein (GO:0006986)	DNAJA1; HSPA1B; HSPA1A
Regulation of cell proliferation (GO:0042127)	TCF7L2; CXCL10; NOTCH1; CDK6; ERBB3; FABP7; POU3 F2; HSPA1B; HSPA1A
Negative regulation of apoptotic process (GO:0043066)	DNAJA1; NOTCH1; SON; ERBB3; ANGPTL4; HSPA1B; HS PA1A
**Down regulated**	
Cholesterol biosynthetic process (GO:0006695)	FDPS; SQLE; MVD; ACAT2; FDFT1
Intermediate filament bundle assembly (GO:0045110)	NEFL; NEFM; NEFH
Anterograde axonal transport (GO:0008089)	FMR1; HSPB1; NEFL; AP3B2
Regulation of steroid biosynthetic process (GO:0050810)	FDPS; SQLE; MVD; FDFT1
Positive regulation of neuron projection development (GO:0010976)	RIMS2; NTRK1; NDRG4; AVIL; STMN2
Microtubule polymerization or depolymerization (GO:0031109)	TPPP3; STMN2; STMN3
Regulation of neurotransmitter secretion (GO:0046928)	RIMS2; FMR1; CPLX1
Axonal transport (GO:0098930)	FMR1; NEFL; AP3B2
Positive regulation of neuron differentiation (GO:0045666)	NTRK1; NDRG4; AVIL; STMN2

**FIGURE 4:**
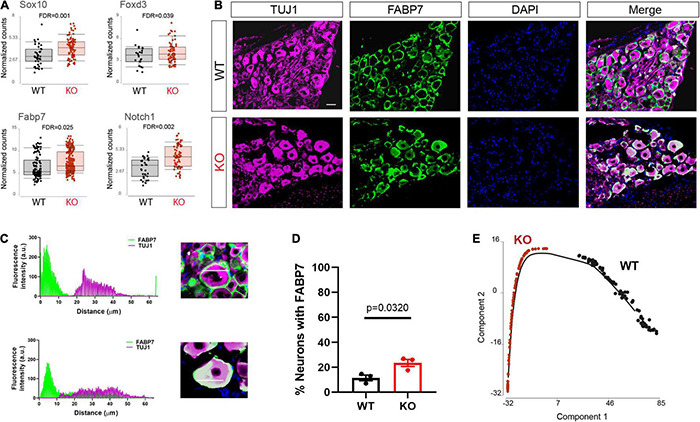
Dysregulated neuronal maturation in *Fmr1* KO DRG. **(A)** Mean expression of the progenitor markers *Sox10*, *Fabp7*, *Notch1*, and *Foxd3* in WT (black) and *Fmr1* KO (red). **(B)** Representative immunofluorescence images of DRG sections from WT and *Fmr1* KO mice co-stained with TUJ1 (magenta) and the SGC marker FABP7 (green). *n* = 3 biologically independent animals. Scale bar: 50 μm. **(C)** Representative images of fluorescence intensity of FABP7/TUJ1 measured across neuron-SGC units (white line in inset image). **(D)** Percentage of neurons with detectable FABP7 expression in WT and *Fmr1* KO. *n* = 3 biologically independent animals, 10–15 cells from each animal were used for measurements. **(E)** Trajectory analysis of small/medium neurons colored by genotype.

Upon differentiation, sensory neurons also normally downregulate glial and progenitor markers. We found that progenitor markers such as *Sox10*, *Fabp7*, *Foxd3*, and *Notch1* were abnormally upregulated in the *Fmr1* KO neurons compared to WT (Figure 4A). We previously showed that *Fabp7* is highly expressed in all SGCs in adult mice (Avraham et al., 2020, 2021b). However, *Fabp7* is also expressed in radial glial cells as well as neuronal progenitors and is critical for neurogenesis in the CNS (Feng et al., 1994; Matsumata et al., 2012). We thus examined by immunofluorescence if FABP7 was upregulated in neurons of *Fmr1* KO DRG compared to WT. As expected, there was minimal spatial overlap of FABP7 and TUJ1 staining in WT, confirming very low neuronal expression of FABP7 in developed sensory neurons (Figures 4B–D). In contrast, overlap of FABP7 and TUJ1 staining was clearly evident in *Fmr1* KO, highlighting a greatly increased number of neurons expressing FABP7 (Figures 4B–D), and validating the scRNAseq data.

To further explore changes in neuronal maturation caused by FMRP loss, we compared the lineage profiles of neurons in *Fmr1* KO and WT, using a differential trajectory map based on “Monocle2.” This analysis can order a set of individual cells along a path/trajectory/lineage, and assign a pseudo-time value to each cell that represents where the cell is along that path. This trajectory analysis further revealed the different developmental stage of neurons in WT vs. *Fmr1* KO (Figure 4E). These results suggest a delayed or aberrant sensory neuronal development process resulting from the loss of FMRP. These observations are consistent with developmental delays suggested in mouse cortical neurons (Tervonen et al., 2009; Saffary and Xie, 2011; Edens et al., 2019), as well as in human neurons and forebrain organoids (Sunamura et al., 2018; Kang et al., 2021).

### Transcriptional Changes in Satellite Glial Cells Caused by Loss of Fragile X Mental Retardation Protein Are Related to Vesicle Organization and Secretion

**FIGURE 5:**
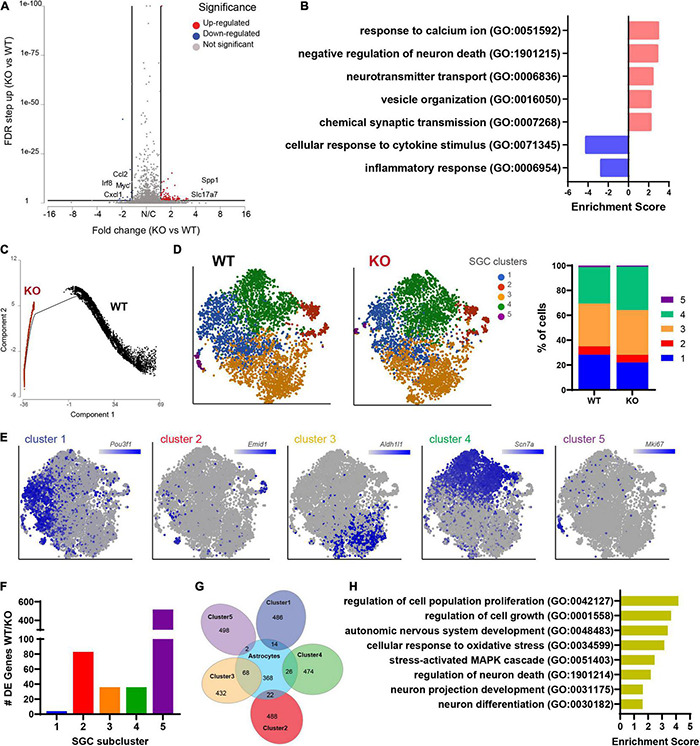
Molecular changes in *Fmr1* KO SGCs are related to vesicle organization and secretion. **(A)** Volcano plot of DE genes in SGCs from *Fmr1* KO compared to WT. **(B)** Enriched pathways in *Fmr1* KO (GO Biological Process) for up-regulated (red) and down-regulated (blue) genes. **(C)** Trajectory analysis of SGCs colored by genotype. **(D)** t-SNE plot of SGC cluster colored by subclusters (unbiased, Graph based clustering) with quantification of the fraction of cells in the different SGC subclusters out of total number of SGC. **(E)** t-SNE overlay with SGC cluster specific genes. **(F)** Quantification of the number of differentially expressed genes in each SGC subcluster in *Fmr1* KO compared to WT (FDR ≤ 0.05, fold-change ≥ 1.5). **(G)** Venn diagram comparing signature genes in SGC subclusters and astrocytes. **(H)** Enriched pathways (GO Biological process) for top differentially upregulated genes in subcluster3 in *Fmr1* KO compared to WT (FDR ≤ 0.05, fold-change ≥ 1.5).

We next examined the transcriptional profile of SGCs in *Fmr1* KO and WT mice. We pooled all cells classified as SGCs (9,917 cells, 5,173 in WT and 4,744 in *Fmr1* KO) and compared the gene expression in *Fmr1* KO and WT SGCs (FDR ≤ 0.05 FC ≥ 1.5). We found 111 genes that were differentially upregulated in *Fmr1* KO SGCs and 19 genes that were downregulated (Figure 5A and Supplementary Table 4). Pathway enrichment analysis (GO biological process) revealed that the upregulated genes were related to calcium signaling, vesicle organization and chemical synaptic transmission (Figure 5B and Table 2). Downregulated pathways were related to response to cytokine stimulus and inflammatory response (Figure 5B and Table 2). These downregulated pathways are consistent with the reduced serum levels of pro-inflammatory chemokines in FXS individuals (Van Dijck et al., 2020). Interestingly, the chemokine *Ccl2* was downregulated in both SGC and macrophages in *Fmr1* KO mice compared to WT (Supplementary Tables 4, 6), and is also reduced in the serum of FXS patients (Van Dijck et al., 2020).

**TABLE 2:** Enriched biological processes (GO 2018) for differentially up and down regulated genes in *Fmr1* KO SGCs.

GO biological process term	Genes
**Up regulated**	
Response to calcium ion (GO:0051592)	TRPC3; SYT1; DPEP1; KCNH1
Negative regulation of neuron death (GO:1901215)	CHGA; UNC5B; VSTM2L; ISL1
Neurotransmitter transport (GO:0006836)	SLC17A6; SLC17A7
Vesicle organization (GO:0016050)	RPH3A; SYT1; VAMP1; DNM1
Chemical synaptic transmission (GO:0007268)	GABBR2; SYT1; PLP1; SLC17A6; SLC17A7; GABRG2
**Down regulated**	
Cellular response to cytokine stimulus (GO:0071345)	MYC; LCN2; CCL2; IRF8; CXCL1
Inflammatory response (GO:0006954)	CYBB; CCL2; CXCL1

We then determine if the SGC subtypes and their similarity to astrocytes we previously characterized (Avraham et al., 2021b) were affected by FMRP loss. Trajectory analysis revealed major differences between WT and *Fmr1* KO SGCs (Figure 5C), that might be indicative of a different developmental process in SGC in the absence of FMRP. Despite these major differences in trajectories, an unbiased clustering of SGCs in WT and *Fmr1* KO revealed similarity in SGC subtype distribution (Figure 5D). Overlay of top marker genes for each SGC cluster paralleled our previous findings of SGC subtypes (Avraham et al., 2021b; Figure 5E), with the exception for an additional cluster 5 in both *Fmr1* KO and WT, which was enriched in proliferation markers such as *Top2a*, *Cdk1*, and *Mki67* (Figure 5E and Supplementary Table 14) and could result from the mice being younger in the current study (4-weeks old), compared to our previous studies using more mature mice (8–12 weeks old). A differential expression analysis between *Fmr1* KO and WT for each SGC cluster revealed larger changes in clusters 2 and 5, moderate changes in clusters 3 and 4 and minor in cluster 1 (FDR ≤ 0.05 FC ≥ 1.5) (Figure 5F). We examined the SGC cluster 3 in more details, since cluster 3 shared the most genes with astrocytes (Figure 5G), consistent with our previous findings (Avraham et al., 2021b). Loss of FMRP in astrocytes induces developmental delays in maturation and elaboration of neuronal dendrites and altered synaptic protein expression (Jacobs et al., 2010; Yang et al., 2012; Wang et al., 2016). Enriched pathways of the differentially expressed genes in cluster 3 in *Fmr1* KO compared to WT suggested a role in neuronal growth and differentiation as well as stress-activated responses (Figure 5H). These results thus further support the notion that SGCs’ role in supporting sensory neuron development might be impaired in *Fmr1* KO.

### Fragile X Mental Retardation Protein Loss Leads to Altered Vesicle Organization in Satellite Glial Cells

**FIGURE 6:**
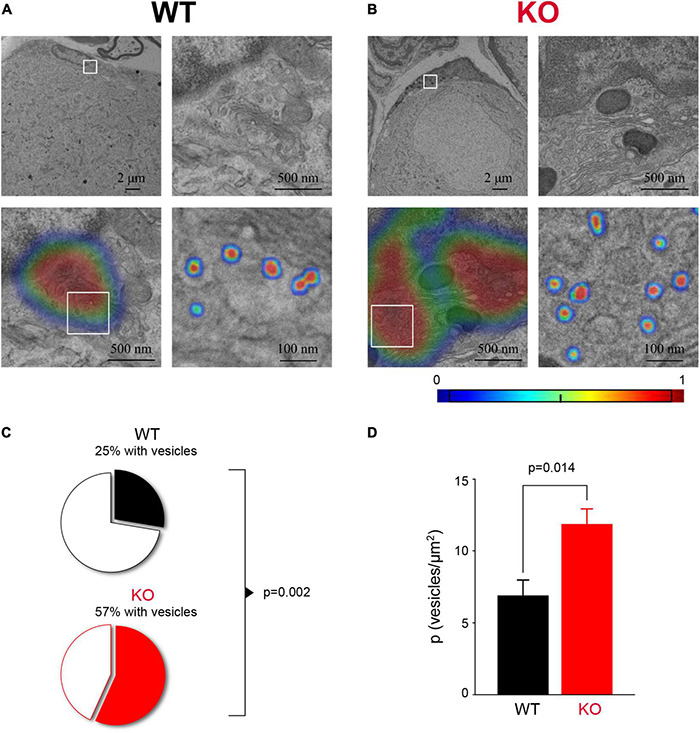
Increased vesicle number in *Fmr1* KO SGC cytoplasm. **(A)** Example of SGC cytoplasmic area next to the SGC nucleus from WT mice (top left) with a boxed area zoomed-in (top, right). The area of interest (bottom left, same area as top right) with the heat-map showing the probability to identify an area with vesicles determined with a gradient-weighted class activation mapping approach. Boxed area is zoomed-in showing segmentation of vesicles using a separate deep learning network (bottom, right). **(B)** The same as panel **(A)**, but for *Fmr1* KO SGCs. **(C)** The proportion of SGCs with detectable vesicles (*P* = 0.0021, Chi-square test). *n* = 4 biologically independent animals. **(D)** Average vesicle density in SGC (*P* = 0.014, KS test). *n* = 4 biologically independent animals.

How SGCs communicate with sensory neurons remains poorly understood. There is evidence that SGCs can secrete factors such as TNFα, ATP, and GABA in certain conditions (Bowery et al., 1976; Hosli and Hosli, 1978; Hanani and Spray, 2020), but whether glial secretion occurs through vesicular release is not well-studied. The transcriptome analysis of SGCs indicated enrichment for genes related to vesicle organization and secretion as one of the most predominant changes in *Fmr1* KO SGCs (Figure 5B). Thus, we sought to verify this observation using ultrastructural measurements. We used TEM to image the SGC cytoplasm at high-resolution and observed multiple vesicular profiles of ∼50 nm diameter in both WT and *Fmr1* KO SGCs (Figures 6A,B). We then applied deep learning network algorithms (Selvaraju et al., 2020) to quantify the number of vesicular profiles in the SGC cytoplasm (Figures 6A,B; see section “Materials and Methods” for details). This analysis revealed a significant increase in the number of vesicles in *Fmr1* KO SGCs cytoplasm compared to WT (Figures 6C,D), validating the scRNAseq data. These results suggest that loss of FMRP causes SGCs to upregulate pathways related to vesicular secretion and thus communication with neurons, which could be a compensation for the disrupted glia-neuron association at the structural level.

## Discussion

Our results revealed major ultra-structural and transcriptional changes caused by loss of FMRP in the peripheral sensory system, including sensory neurons and their enveloping SGCs. Our analyses suggest structural disruption of sensory neuron-SGC association in the absence of FMRP, which is accompanied by aberrant transcriptional changes in pathways involved in vesicle secretion and neuron-glia communication. These abnormalities are also accompanied by transcriptional changes indicative of delayed/altered maturation of neurons and SGCs. Given the well-established role for SGCs in regulating sensory neurons functions (Hanani and Spray, 2020), and the similarity between human and mouse SGCs (Avraham et al., 2021a), the structural and functional disruption of neuron-SGC communication may have important contribution to sensory dysfunctions in FXS.

Many aspects of neuronal function in the DRG are regulated by neuron-SGC interactions (Hanani and Spray, 2020). Thus, the disruption of neuron-glia association and altered vesicular communication could contribute to sensory defects *via* several mechanisms. First, SGC share functional similarities with astrocytes (Avraham et al., 2020; Hanani and Spray, 2020; Hanani and Verkhratsky, 2021) and loss of FMRP in astrocytes induces developmental delays in maturation and elaboration of hippocampal neuron dendrites and altered synaptic protein expression (Jacobs et al., 2010; Yang et al., 2012; Wang et al., 2016). Our results reveal that, similarly to central neurons, peripheral sensory neurons in *Fmr1* KO have a markedly delayed developmental trajectory. Interestingly, we observed that SGC cluster 3, which shares the most similarities with astrocytes, is altered by the loss of FMRP, with enriched pathways related to neuronal differentiation and development. Disruption of SGCs-neuron association and communication, if present during early development, could thus contribute to the developmental delay in maturation of sensory neurons in the absence of FMRP. Whether all sensory neuron subtypes present aberrant interaction with their surrounding SGC in the *Fmr1* KO mouse model, or specific subtypes are preferentially affected remains to be elucidated. This will be interesting to pursue in future studies, as various subtypes of sensory neurons encode different sensory modalities, such as pain and touch, that are affected in FXS individuals.

Second, SGCs are well-known to regulate neural excitability in normal conditions and to lead to pathological neuronal hyperexcitability in many pain syndromes (Hanani and Spray, 2020). SGCs can regulate neuronal excitability *via* ATP release (Du et al., 2011; Yousuf et al., 2011). Also, both sensory neurons and SGCs express glutamate receptors (Kung et al., 2013), although whether SGCs release glutamate has not been established definitively. Interestingly, it has long been known that SGCs can uptake and release GABA in a calcium-dependent manner (Minchin and Iversen, 1974; Minchin, 1975; Bowery et al., 1976; Hosli and Hosli, 1978) and that sensory neurons express GABA_A_ and GABA_B_ receptors (Labrakakis et al., 2003; Du et al., 2017). A recent study found that a subpopulation of SGCs in the DRG express GABA, the Bestrophin-1 (Best1), a Ca^2+^ dependent chloride channel, and enzymes of the putrescine pathway responsible for an alternative route of GABA biosynthesis, suggesting that SGCs may be the source of endogenous GABA released in the DRG (Vargas-Parada et al., 2021). Importantly, SGC-mediated inhibition is dependent on a close association between neurons and SGCs (Shoji et al., 2010; Vargas-Parada et al., 2021). Thus, structural disruption of neuron-SGCs association, as we observed in *Fmr1* KO mice, can contribute to sensory neuron hyperexcitability, in part, by reducing effects of GABA release from SGCs. Future studies will be needed to define the complex secretome of SGCs, which may include ATP, GABA, and glutamate.

Third, signaling pathways which are down-regulated in SGCs of *Fmr1* KO mice are primarily linked to inflammatory and cytokine response. This is consistent with findings of cytokine imbalance in FXS individuals (Van Dijck et al., 2020), children with autism (Yu et al., 2020) and the growing evidence that neuro-inflammatory mechanisms may contribute to the pathogenesis of FXS as well as other autism-associated disorders (Di Marco et al., 2016; Yu et al., 2020). This observation is particularly intriguing in a view of a growing number of reports that cytokines may specifically interact with neuronal ion channels regulating excitability (Viviani et al., 2007; Schafers and Sorkin, 2008). Pro-inflammatory cytokines are known to modulates L- and N-type Ca^2+^channels, Na^+^-channels, and GABA_A_ receptors, and to regulate trafficking of AMPA and GABA_A_ receptors in central neurons as well as glutamate uptake by glial transporters (Viviani et al., 2007; Schafers and Sorkin, 2008). Thus, dysregulation of the inflammatory and cytokine response in SGCs in *Fmr1* KO mice could contribute to sensory neuronal dysfunction *via* multiple mechanisms.

In summary, our results uncover major defects in neuron-SGCs association and communication, suggesting that manipulation of SGCs function maybe a viable new strategy to normalize function of peripheral sensory neurons in FXS.

## Materials and Methods

### Animals

*Fmr1* KO and WT control mice on FVB background were obtained from The Jackson Laboratory. Male mice (28–30-day old) were used. All animal procedures were in compliance with the NIH Guide for the Care and Use of Laboratory Animals and conformed to Washington University Animal Studies Committee guidelines.

### Transmission Electron Microscopy

Mice were perfused with 2.5% glutaraldehyde and 4% paraformaldehyde in 0.1 M Cacodylate buffer, followed by post fix. A secondary fix was done with 1% osmium tetroxide. For TEM, tissue was dehydrated with ethanol and embedded with spurr’s resin. Thin sections (70 nm) were mounted on mesh grids and stained with 8% uranyl acetate followed by Sato’s lead stain. Sections were imaged on a Jeol (JEM-1400) electron microscope and acquired with an AMT V601 digital camera (Washington University Center for Cellular Imaging). For measurements of the number of neurons with distant glia, neurons were counted as 0 (no distant glia) or 1 (obvious distant glia). To quantify the distance between the neuron outer membrane and the SGC, we performed 3 measurements per neuron, where we detected the largest visible gaps between the neuron membrane and the SGC membrane. Neuron’s diameter was measured at the neuron’s largest point across. Neuron circularity was measured as a ratio between major axis (X) and minor axis (Y) (1 = circular, 1 < elliptic). Neuronal area was measured by manually tracing the neuron outer membrane. For all measurements ImageJ software was used.

### Vesicle Detection in Transmission Electron Microscopy Sections With Deep Learning Neural Networks

Customized two-step deep learning algorithm utilizing GoogLeNet network was developed to detect and count SGC vesicles in TEM sections. First, we used the gradient-weighted class activation mapping (Grad-CAM) technique to scan the images to produce a localization map identifying regions of interest with vesicles in the image. We then used a second trained network to identify and segment individual vesicles (Selvaraju et al., 2020). The first network was trained with 2,671 pre-label images, 20% of the data was randomly assigned to the validation group. Network was trained using a mini-batch of 25 and a maximum epoch of 500, obtaining an accuracy of over 99% in the validation group. In order to prevent over fitting, we perform data augmentation by randomly resizing by 10% and reflecting the image. The second network was trained with 4,425 pre-label images, 20% of the data was randomly assigned to the validation group. This network was trained using a mini-batch of 100 and a maximum epoch of 1,000 obtaining an accuracy of 98.2% in the validation group. Given the small number of pixels that define a vesicle, data augmentation was prone to generate image artifacts that reduce the performance of the network. To overcome this problem, we limited the data augmentation to 5% resizing and used reflection and pixel shift in addition. The vesicle density was determined using a clustering algorithm and the area was defined as the smallest convex set that contains all the vesicles (convex hull area).

### Single Cell RNAseq

DRG collected from 3 mice from each genotype for two biological replicates were dissociated into single cell suspension as describe (Avraham et al., 2020, 2021b). Cells were then washed in HBSS + Hepes + 0.1%BSA solution, passed through a 70-micron cell strainer. Hoechst dye was added to distinguish live cells from debris and cells were FACS sorted using MoFlo HTS with Cyclone (Beckman Coulter, Indianapolis, IN). Sorted cells were washed in HBSS + Hepes + 0.1%BSA solution and manually counted using hemocytometer. Solution was adjusted to a concentration of 500 cell/microliter and loaded on the 10X Chromium system. Single-cell RNA-Seq libraries were prepared using GemCode Single-Cell 3’ Gel Bead and Library Kit (10x Genomics). A digital expression matrix was obtained using 10X’s CellRanger pipeline (Build version 2.1.0) (Washington University Genome Technology Access Center). Quantification and statistical analysis were done with Partek Flow package (Build version 9.0.20.0417).

#### Filtering Criteria

Low quality cells and potential doublets were filtered out from analysis using the following parameters; total reads per cell: 600–15,000, expressed genes per cell: 500–4,000, mitochondrial reads < 10%. A noise reduction was applied to remove low expressing genes = 1 count. Counts were normalized and presented in logarithmic scale in CPM (count per million) approach. An unbiased clustering (graph based clustering) was done and presented as t-SNE (t-distributed stochastic neighbor embedding) plot, using a dimensional reduction algorithm that shows groups of similar cells as clusters on a scatter plot. Differential gene expression analysis performed using an ANOVA model; a gene is considered differentially expressed (DE) if it has a false discovery rate (FDR) step-up (*p*-value adjusted). *p* ≤ 0.05 and a fold-change ≥±1.5. Lowering the cutoff of fold-change down to ≥± 1.3, result in similar gene sets and pathway analysis. The data was subsequently analyzed for enrichment of GO terms using Partek flow pathway analysis. Partek was also used to generate figures for t-SNE plots using a statistical method for visualizing high-dimensional data by giving each data point a location in a two-dimensional map.

#### Trajectory Analysis

We performed a differential trajectory mapping using “Monocle2” with standard settings. The algorithm orders a set of individual cells along a path/trajectory/lineage, and assigns a pseudo-time value to each cell that represents where the cell is along that path. This method identifies intermediate states during a biological process as well as bifurcation between two alternative cellular fates.

### Immunohistochemistry

After isolation of DRG, tissue was fixed using 4% paraformaldehyde for 1 h at room temperature. Tissue was then washed in PBS and cryoprotected using 30% sucrose solution at 4^°^C overnight. Next, the tissue was embedded in O.C.T., frozen, and mounted for cryosectioning. All frozen sections were cut to a width of 12 μm for subsequent staining. Slides were washed 3× in PBS and then blocked for in solution containing 10% goat serum in 0.2% Triton-PBS for 1 h. Next, sections were incubated overnight in blocking solution containing primary antibody. The next day, sections were washed 3× with PBS and then incubated in blocking solution containing a secondary antibody for 1 h at room temperature. Finally, sections were washed 3× with PBS and mounted using ProLong Gold antifade (Thermo Fisher Scientific). For immunofluorescence on cultured cells, DRG cells were cultured on 100 μg/ml poly-D-lysine coated cover slips for 4 days in neurobasal media. Cells were then fixed using 4% paraformaldehyde for 20 min at room temperature. Cover slips were incubated for 1 h in 0.1% Triton-PBS containing primary antibody washed 3× with PBS and then incubated in 0.1% Triton-PBS solution containing a secondary antibody for 1 h at room temperature followed by 3 washes in PBS. Images were acquired at 10× or 20× using a Nikon TE2000E inverted microscope and images were analyzed using Nikon Elements. To determine the cleaved caspase staining area, a binary was generated to fit the positive signal, and positive staining area was measured. We noticed that the cleaved caspase signal was mainly in neuronal cells, therefore that area was internally normalized to TUJ1 positive staining area (ImageJ). To measure the number of cleaved caspase 3 positive neurons we manually selected all neurons (TUJ1 positive cells) with signal of cleaved caspase-3 above background (ImageJ). To measure the intensity of FABP7 signal in neurons, a 60 μm line was drawn across the neuron. Fluorescence intensity for FABP7 and TUJ1 was measured along the line (Nikon Elements).

Antibodies were as follow: Tubb3 (TUJ1) antibody (BioLegend catalog #802001, RRID:AB_291637), FABP7 (Thermo Fisher Scientific Cat# PA5-24949, RRID:AB_2542449), cleaved caspase 3 (CST Cat# 9664, RRID:AB_2070042).

### RNA Isolation and Quantitative PCR

DRG and nerves were lysed and total RNA was extracted using Trizol reagent (Thermo Fisher Scientific, Cat# 15596026).). Next, RNA concentration was determined using a NanoDrop 2000 (Thermo Fisher Scientific). First strand synthesis was then performed using the High Capacity cDNA Reverse Transcription kit (Applied Biosystems). Quantitative PCR was performed using PowerUp SYBR Green master mix (Thermo Fisher Scientific, Cat# a25742) using 5 ng of cDNA per reaction. Plates were run on a QuantStudio 6 Flex and analyzed in Microsoft Excel. The average Ct value from three technical replicates was averaged and normalized to the internal control Rpl13a. All primer sequences were obtained from PrimerBank and product size validated using agarose gel electrophoresis.

Rpl13a (PrimerBank ID 334688867c2) Forward Primer AGCCTACCAGAAAGTTTGCTTAC Reverse Primer GCTTCTTCTTCCGATAGTGCATC

Tubb3 (PrimerBank ID12963615a1) Forward Primer TAGACCCCAGCGGCAACTA

Reverse Primer GTTCCAGGTTCCAAGTCCACC

Nefl1 (PrimerBank ID 200038a1) Forward Primer CCGTACTTTTCGACCTCCTACA

Reverse Primer CTTGTGTGCGGATAGACTTGAG

Stmn2 (PrimerBank ID 118130361c1) Forward Primer CAGAGGAGCGAAGAAAGTCTCA

Reverse Primer CTAGATTAGCCTCACGGTTTTCC

### Statistical Analysis

Data are presented as means ± SEM. Student’s paired or unpaired *t*-test, KS test or Chi-square test were used for statistical analysis as appropriate; significance was set as *p* < 0.05. The n was number of cells tested, unless otherwise stated. All statistical values and tests used in each experiment are given in Supplementary Table 1 for each panel, as well as in each figure legend.

## Data Availability Statement

The datasets generated in this study can be found in online repositories. The names of the repository/repositories and accession number(s) can be found below: https://www.ncbi.nlm. nih.gov/search/all/?term=GSE176449.

## Ethics Statement

All animal procedures were reviewed and approved by the Washington University School of Medicine Institutional Animal Care and Use Committee (IACUC) under protocol A-3381-01. All experiments were performed in accordance with the relevant guidelines and regulations. All experimental protocols involving mice were approved by the Washington University School of Medicine (protocol #21-0104 and #20-0173). Mice were housed and cared for in the Washington University School of Medicine animal care facility. This facility is accredited by the Association for Assessment and Accreditation of Laboratory Animal Care (AALAC) and conforms to the PHS guidelines for Animal Care. Accreditation - 7/18/97, USDA Accreditation: Registration #43-R-008.

## Author Contributions

OA, P-YD, VC, and VK conceived and designed the experiments. P-YD and OA performed the experiments. DM contributed deep learning analysis tools. P-YD, OA, and DM performed data analysis. VC and VK secured the funding. All authors wrote the manuscript and approved the submitted version.

## Conflict of Interest

The authors declare that the research was conducted in the absence of any commercial or financial relationships that could be construed as a potential conflict of interest.

## Publisher’s Note

All claims expressed in this article are solely those of the authors and do not necessarily represent those of their affiliated organizations, or those of the publisher, the editors and the reviewers. Any product that may be evaluated in this article, or claim that may be made by its manufacturer, is not guaranteed or endorsed by the publisher.

## Supplementary Material

Supplementary Table 1 Data values and statistical analyses. Columns represent (from left to right): figure/panel number; experimental conditions; mean values and SEM; number of samples, statistical test used for comparison; *P*-value resulting from the statistical comparison.

Supplementary Table 2 Differentially expressed (DE) genes per cell type relative to all other cell clusters (ANOVA Fold change > 1.5).

Supplementary Table 3 Differentially expressed (DE) genes in *Fmr1* KO small/medium neurons (FDR < 0.05, Fold change > 1.5).

Supplementary Table 4 Differentially expressed (DE) genes in *Fmr1* KO SGCs (FDR < 0.05, Fold change > 1.5).

Supplementary Tables 5–13 Differentially expressed (DE) genes in *Fmr1* KO for endothelial cells, macrophages, mesenchymal endoneural, mesenchymal epineural, pericytes, Schwann cells, smooth muscle cells, large neurons and T-cells (FDR < 0.05, Fold change > 1.5).

Supplementary Table 14 Differentially expressed (DE) genes per SGC cluster relative to all other SGC clusters (ANOVA Fold change > 1.5). Shared genes with astrocytes for each SGC subcluster and DE genes in *Fmr1* KO SGC for cluster 3.
